# A Simple Procedure to Assess Limit of Detection for Multisensor Systems

**DOI:** 10.3390/s19061359

**Published:** 2019-03-18

**Authors:** Ekaterina Oleneva, Maria Khaydukova, Julia Ashina, Irina Yaroshenko, Igor Jahatspanian, Andrey Legin, Dmitry Kirsanov

**Affiliations:** 1 Institute of Chemistry, Saint-Petersburg State University, Universitetskaya emb., 7-9-11, 199304 Saint-Petersburg, Russia; ekaterina.oleneva@inbox.ru (E.O.); khaydukova.m@gmail.com (M.K.); ashina.julia91@gmail.com (J.A.); irina.s.yaroshenko@gmail.com (I.Y.); andrey.legin@gmail.com (A.L.); 2 Laboratory of Artificial Sensory Systems, ITMO University, Kronverksky pr., 49, 197101 Saint-Petersburg, Russia; drjie@mail.ru

**Keywords:** limit of detection, multisensor systems, first-order multivariate calibration, electronic tongue, potentiometric sensors

## Abstract

Currently, there are no established procedures for limit of detection (LOD) evaluation in multisensor system studies, which complicates their correct comparison with other analytical techniques and hinders further development of the method. In this study we propose a simple and visually comprehensible approach for LOD estimation in multisensor analysis. The suggested approach is based on the assessment of evolution of mean relative error values in calibration series with growing analyte concentration. The LOD value is estimated as the concentration starting from which MRE values become stable from sample to sample. This intuitive procedure was successfully tested with a variety of real data from potentiometric multisensor systems.

## 1. Introduction

Multisensor system is an analytical device which is based on arrays of cross-sensitive chemical sensors combined with multivariate data processing tools [1]. These systems can be successfully applied for a variety of analytical tasks [2,3] including such non-trivial examples e.g., toxicity assessment [4] or taste evaluation [5]. Various multisensor systems are an effective tool for the improvement of analytical selectivity in complex multicomponent samples where individual high-selective sensors are hardly convenient. In spite of the large progress in this field through the last decade [6,7], there are still certain points left unattended by researchers. The absence of formal figures of merit for multisensor systems (like e.g., LOD and selectivity) definitely hinders the development of the general multisensor concept and its integration into a common laboratory practice. The existing inconsistency in estimation of analytical characteristics of multisensor systems complicates the comparison of this approach with other analytical methods. It would be beneficial for the researches to have established and agreed definitions for sensitivity, selectivity, and limit of detection in multisensor studies when it is applicable (for example, when an integral parameter is evaluated—such as water toxicity or taste attributes—the LOD concept itself is not defined and cannot be used for a system characterization). 

Limit of detection is one of the key characteristics of any analytical method which allows for comparison between different analytical techniques and for choosing the most efficient and suitable one for particular analytical task. The LOD value indicates the lowest quantity of an analyte which can be distinguished from the absence of that substance with a certain confidence level [8]. This parameter is widely used in analytical chemistry, but the limit of quantification (LOQ) might also be useful as a figure of merit, which defines the lowest reliably detected analyte concentration. Nevertheless, these two values can be easily converted into each other, so here and after only LOD will be taken into consideration. A modern formula for LOD value estimation in case of univariate calibration (LOD_U_)—when certain sample attribute (i.e., concentration) is associated with one particular signal value (i.e., peak intensity at a certain wavelength in a spectrum)—is recommended by IUPAC [8]:

(1) $${\mathrm{LOD}}_{U}=\frac{3.3s_{y/x}}{A}\sqrt{1+h_{0}+\frac{1}{I}}$$

where s_y/x_ is a residual standard deviation, A is a slope of a univariate calibration graph, I is a total number of samples used for the calibration, h_0_ is a leverage for the blank sample. This general formula can be specified for different analytical methods for the sake of usability. In case of a single chemical sensor LOD value should be estimated as a point of intersection of extrapolated linear regions of the calibration curve [9,10]. If the comparison with other analytical methods is required, LOD estimation as a standard deviation of the potentiometric signal noise s_bi_ multiplied by three is more consistent [11]:

(2) $$x_{l}=\frac{3s_{\mathrm{bi}}}{A}$$

Evaluation of LOD in case of multivariate calibration represents a non-trivial task due to the complexity of obtained data—concentration value of a single analyte can be associated with a row vector of signal values (first-order data) or even with a signal matrix (second-order data). In these cases, the representation of a calibration curve as a function of signal intensity from concentration is hardly possible. Multisensor systems generally provide their response as a first-order data set and multivariate calibration models are required to relate the vector of responses from all sensors with particular sample parameter of interest, e.g., concentration of an analyte. Obviously, classical univariate approach for LOD estimation cannot be directly applied to this kind of data. 

Currently, there is no officially accepted procedure for LOD estimation in multivariate measurements. However, intense development of analytical methods employing multivariate modelling of first and second-order data makes this problem vital and different approaches to multivariate LOD definition and calculation have been presented [12,13,14,15,16]. The majority of them have been suggested for spectroscopic methods, for example, the concept of net analyte signal (NAS), developed by Lorber [17]. The main idea of NAS concept is to find, for an analyte under study, its own contribution to the entire spectral data set, which is orthogonal to the spectra of other mixture components, and to use this part of spectra for further analyte quantification and signal-to-noise ratio, LOD and other figures of merit estimation. The NAS approach applicability for electrochemical data was also demonstrated by Burgués et al. [18] for mixed-oxide semiconductor sensors for CO detection in the air. However, in cases of multisensor systems with cross-sensitive sensors, developed for simultaneous quantification of several analytes in different concentration ranges, the underlying condition of data homoscedasticity is not always fulfilled. Heteroscedastic data are very typical for this kind of measurements and can be used for further regression model building without any concern. 

The group of Olivieri [19,20,21,22,23] has proposed the universal approach for multivariate LOD interval estimation, potentially applicable to any type of analytical measurements and any data processing method. Nevertheless, its application for multisensor systems data (that are mainly electrochemical) is not straightforward, as it is for spectroscopic data. The main difficulty is in different nature of variables: while in spectroscopy the variance of instrumental signal can be obtained by simple replicate analysis due to the equivalence of all wavelengths, in multisensor systems each variable or sensor can be unique by its membrane composition and its pattern of response on the same analyte due to the cross-sensitivity and different way of “ionophore-target ion” interaction. Thus, the contribution of each sensor has to be taken into account in the total variance of instrumental signal estimation. In the recent work of Burgués et al. [24], devoted to the multivariate LOD calculation for the same type of sensors, the adaptation of Olivieri method for mixed-oxide sensors was demonstrated. Nonetheless, the approach suggested in this paper has the same drawback as Olivieri method: the estimation of analytical signal standard deviation is still required. Also, the authors had two types of sensors (7 units of each type) and described them separately; in case of multisensor systems, all sensors of different types have to be entirely taken into consideration.

The common disadvantage of the majority of proposed LOD estimation approaches is in the complexity of involved calculations. While it is surely important to have methods which comply with the existing formalism and obey strict definitions, such fundamentality is not usually required for applied analytical tasks, when a chemist or an engineer has to rank available analytical methods as potentially suitable or not for the given task. A fast, simple, and intuitive method of LOD estimation would be a good helper at this step. The pseudounivariate LOD approach [19] seems to be relatively simple, but, as it was shown by the authors themselves, in 3 of 6 examples of LOD calculation for real data sets, the results obtained with this simplified formula exceed the LOD interval values in 3–6 times. The question which one should be taken as a true LOD value is disputable.

The purpose of this study was to propose a simple and intuitive methodology for LOD estimation in multisensor studies. The idea of the approach is based on the mean relative error evolution and it does not require any sophisticated calculations. Moreover, the proposed approach allows for visualization of the multisensor system performance in different regions of the analyte concentration range. Obviously, the obtained LOD value is not theoretically strict, but it can give an idea about multisensor system applicability to a given task. Certainly, this value can be defined more precisely by any other fundamentally justified approach if required. 

## 2. Theory

### 2.1. LOD Interval Calculation

**Figure 1:**
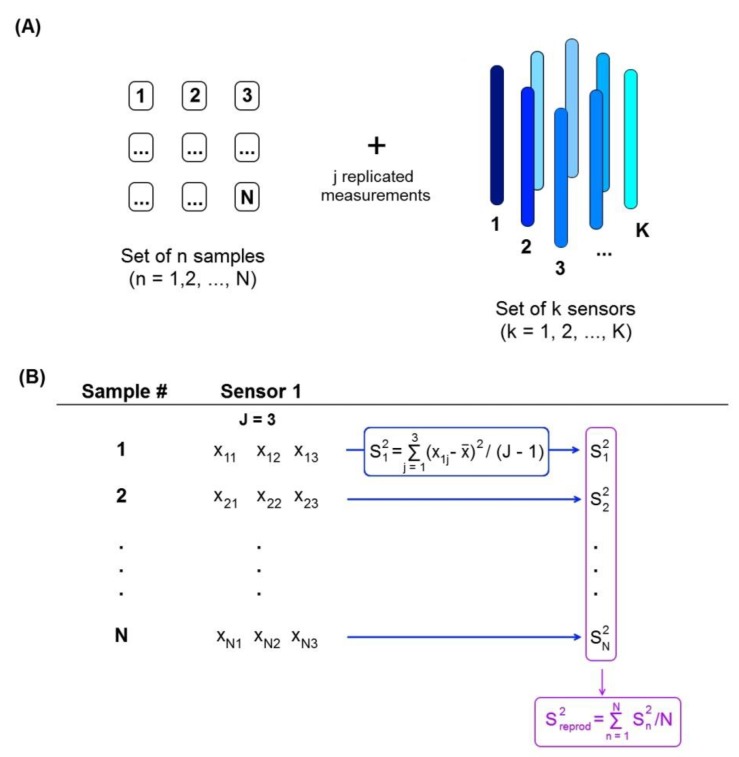
Schematic representation of the reproducibility variance calculation. (**A**) A sample set contains N samples, each of them is measured J times by a multisensor system with K sensors. (**B**) Step-by-step calculation of reproducibility variance shown for the sensor #1.

In this section, the procedure suggested in the work of Olivieri [19] is adapted to the data from potentiometric multisensor system as a typical example of such instruments. According to that study, it is more consistent to calculate the LOD interval instead of one value to avoid dependence of the LOD value on the sample analytical matrix, which is not constant in case of multivariate systems. The formula for LOD_min_ and LOD_max_ are given below:

(3) $${\mathrm{LOD}}_{\min}=3.3{\left[{\mathrm{SEN}}^{-2}\mathrm{var}\left(x\right)\left(1+h_{0\min}\right)+{\;h}_{0\min}\mathrm{var}\left(y_{\mathrm{cal}}\right)\right]}^{1/2}$$

(4) $${\mathrm{LOD}}_{\max}=3.3{\left[{\mathrm{SEN}}^{-2}\mathrm{var}\left(x\right)\left(1+h_{0\max}\right)+{\;h}_{0\max}\mathrm{var}\left(y_{\mathrm{cal}}\right)\right]}^{1/2},$$

where SEN is the sensitivity (inversed vector of regression coefficients), var(x) is the variance in instrumental signals, h_0_ is the estimated blank sample leverage (minimal or maximal, respectively), and var(y_cal_) is the variance of the analyte concentrations in the calibration set. If the estimation of sensitivity of a multivariate regression model and of the var(y_cal_) seem to be easy, the evaluation of the variance in instrumental signals poses a problem. In spectroscopic measurements, the var(x) can be estimated by replicate analysis. In case of electrochemical data obtained with a multisensor system, this approach is not so evident because of the necessity of including the individual contribution of each sensor into the final var(x) estimation. This aspect can be taken into account by applying the formula of experiment reproducibility variance S^2^_reprod_ for each sensor from an array (Figure 1):

(5) $$S_{\mathrm{reprod},k}^{2}=\frac{\sum_{n=1}^{N}\sum_{j=1}^{J}{\left(x_{\mathrm{nj}}-\bar{x}\right)}^{2}}{N\;\left(J-1\right)}$$

where x_nj_ is a response value of a sensor k obtained for n^th^ sample and j^th^ replics, $\bar{x}$ is an average instrumental response for all J repeated measurements, N is a number of samples, and J is the number of repeated measurements.

This formula allows for obtaining a single value of variance which characterizes a sensor’s ability to give reproducible results. Nevertheless, it can be used only for homoscedastic data. To verify the homogeneity of the data, the Cochran’s C test [25] can be applied in a column-wise mode. This test detects if the maximal value of the reproducibility variance does not exceed the Cochran’s C statistic for the same number of samples and repeated measurements [26]:

(6) $$C_{\mathrm{nj}}=\frac{\max(S_{n}^{2})}{\sum_{n=1}^{N}S_{n}^{2}}$$

where C_nj_ is a Cochran’s C statistic for n samples and j repeated measurements, S^2^_n_—reproducibility variance for each sample. C_nj_ value for any number of samples can be approximated by Fischer’s distribution [27]:

(7) $$C_{\mathrm{nj}}={\left[1+\frac{n-1}{F_{c}\left(\frac{\alpha }{N},\left(j-1\right),\left(n-1\right)\left(j-1\right)\right)}\right]}^{-1},$$

where a is a significance level, F_c_—critical value of Fisher’s F ratio (available from the tables or calculated by computer software, e.g., Microsoft Excel).

The total variance of analytical signal for the system, var(x), represents a sum of all calculated S^2^_reprod_:

(8) $$\mathrm{var}\left(x\right)=\sum_{k=1}^{K}S_{\mathrm{reprod},k}^{2},$$

The C_nj_ value, calculated for each sensor, can be used as an exclusion criterion either for the samples (they can be excluded from the entire data set until the Cochran’s C test is passed for all sensors in the array), or for the sensors, if the number of possibly excluded samples seems to be significant. In the latter case, it can be an indication of inappropriate state of the sensor (deterioration, etc.). Nevertheless, when the number of sensors or samples is large, such rejections can be cumbersome and excessive. Thus, for certain data sets this approach is hardly applicable (see Results section). In this situation, the pseudounivariate LOD estimation can be used instead of the LOD interval:

(9) $${\mathrm{LOD}}_{\mathrm{pu}}=3.3\cdot s_{\mathrm{pu}}^{-1}\;\left[\left(1+h_{0\min}+\frac{1}{I}\right){\mathrm{var}}_{\mathrm{pu}}\right],$$

where s_pu_ is the slope of the “measured vs. predicted” line, var_pu_ is the variance of the regression residuals. 

In case of potentiometric multisensor systems, this fundamental approach can be applied with certain limitations, because the condition of data homoscedasticity is not always fulfilled for electrochemical measurements. The described procedure allows for choosing the samples with homoscedastic sensor responses, but in some situations it leads to the undesirable loss of information and significant reduction of number of samples in the data set. 

### 2.2. Mean Relative Error Evolution Approach

A method proposed in this study is based on the idea of visual representation of mean relative error (MRE) increment evolution dependent on an analyte concentration. Basically, MRE is calculated by the formula given below:

(10) $$\mathrm{MRE}=\frac{\left|M-P\right|}{M},$$

where M is measured value (taken as actual), P—predicted value.

MRE describes the difference between the measured analyte concentration and the predicted one, related to the actual value to avoid the effect of different concentration magnitude on the borders of the concentration range. Obviously, for analyte concentrations lower than the LOD, average MRE has to be high, because an instrument cannot properly determine such a small amount of the analyte. The idea is to find the analyte concentration, starting from which the average MRE value significantly decreases, according to the following procedure:

- sort the column with measured analyte concentration y_n_ in ascending order (y_n_ becomes $\tilde{y_{n}}$);
- calculate MRE for each sample;
- average MRE values and the measured concentrations for the first n samples (with the lowest concentration). n = 2 is the most convenient choice. Than use step by step the following formula:
  (11) $${\mathrm{MRE}}_{n^{\prime }}=\frac{1}{n^{\prime }}\sum_{n=2}^{n^{\prime }}\frac{\left|{\tilde{y}}_{n\;\mathrm{meas}}-{\tilde{y}}_{n\;\mathrm{pred}}\right|}{{\tilde{y}}_{n\;\mathrm{meas}}},$$

where n′ is a number of samples for which MRE is averaged (n′ = 2, 3, …, N).

A plot of averaged MRE against averaged concentrations can be used for visualization of a measurement system performance on various concentration intervals. In order to estimate a LOD value, the next step should be added:

- calculate the increment of averaged MRE as Δ<MRE> = |MREn+1′ − MREn′|.

Finally, averaged MRE increment absolute values should be plotted as a function of averaged concentrations. Thus, a LOD for the given analyte is a concentration, starting from which the Δ<MRE> value fluctuations around zero are equal or less than 1% (see Results and Discussion section). Depending on the task and on the desired accuracy level of LOD estimation, the appropriate Δ<MRE> fluctuations magnitude can be varied. The higher is it, the more optimistic is the LOD estimation.

The simplicity of this approach represents its main advantage: only two parameters are required to have an idea about a multisensor system LOD value. The first parameter is known a priori before the multivariate model building, and it includes such uncertainty sources as the analyte signal variance, and the second is the result of the analyte concentration prediction, taking into account the model sensitivity. Besides, since no variance of analytical signal is directly included in the LOD calculations, there is no need to fulfill the condition about data homoscedasticity.

## 3. Experimental

### 3.1. Multisensor Systems

To estimate the LOD values, the electrochemical data from three different potentiometric multisensor systems have been taken (Table 1). 

The first multisensor system has been developed for Ca^2+^ and Mg^2+^ quantification in their aqueous mixtures with Na^+^. The detailed information about membranes preparation and potentiometric measurements is available in Supplementary Materials (S2). To demonstrate the different approaches of the LOD estimation, only the data with Ca^2+^ as an analyte were taken into account.

The second multisensor system was designed for simultaneous quantification of rare earth metals (La, Y, Gd) in complex mixtures [28]; in this work only La^3+^ was taken as an analyte. The third multisensor system has been developed for the determination and quantification of urine ionic components [29]. From 12 identified compounds, we took the data for two of them: Na^+^, Cl^−^. The precise concentration of urine components was measured by capillary electrophoresis as a reference method. 

### 3.2. Data Processing

Partial Least Squares (PLS) algorithm was employed to build the regression models for each data set [30]. PLS models were computed with The Unscrambler^®^ 9.7 (CAMO Software AS, Norway). The models were validated using the full cross-validation (FCV). This validation type was chosen in order to unify the estimation approach for all studied data sets and to avoid the variability caused by possible different choice of the test sets induced by different size of the data. During this procedure, all samples from the data set employed for the model building, are one by one excluded from the calibration set, and used as a test sample. The validation results are described by the root-mean square error of cross-validation (RMSECV):

(12) $$\mathrm{RMSECV}=\sqrt{\sum_{n=1}^{N}\frac{{\left(y_{n\;\mathrm{pred}}-{\;y}_{n\;\mathrm{meas}}\right)}^{2}}{N}}\;,$$

where y_npred_ is predicted analyte concentration in the sample n, y_nmeas_ is a measured analyte concentration in the sample n, N is a number of samples in the data set.

MRE calculations and further LOD estimation were performed using MATLAB R2017a (The Mathworks, USA). A script with the applied function is available in Supplementary Materials (S1).

## 4. Results and Discussion

### 4.1. Multisensor System for Ca^2+^ Quantification in Ca/Mg/Na Mixture

The experimental data (40 samples × 10 sensors) were pretreated according to the procedure detailed before in order to estimate the variance of instrumental signal (Figure 1B). Three samples were excluded to assure the homoscedasticity of the data. Var(y_cal_) was estimated by replication and was equal to 8 × 10^−9^ mol^2^/L^2^. The PLS model for prediction of Ca^2+^ content was built based on the data set and optimal number of latent variables (LV) was equal to 4. Equations (3), (4), and (9) were applied for LOD interval and LOD_pu_ calculation. The results are shown in the Table 2, RMSECV and LOD values are given in mol/L units. LOD_pu_ value, as it usually occurs [14], is larger than the calculated LOD_max_, but all three values are of the same order and do not contradict each other.

**Figure 2:**
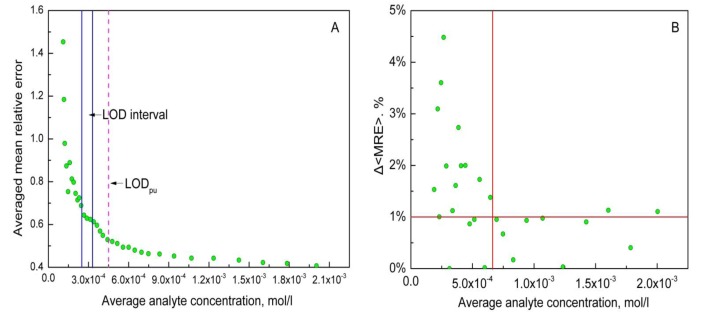
Comparison of calculated and estimated LOD (Ca^2+^) values for the Ca/Mg/Na multisensor system. (**A**): blue rectangle corresponds to the calculated LOD interval, magenta dash line—to the LOD_pu_ value. (**B**): the concentration region corresponding to the Δ<MRE> from 0 to 5% is shown. The horizontal red line indicates the maximal acceptable magnitude of Δ<MRE> fluctuations around zero. The vertical red line indicates the LOD value.

Results obtained with mean relative error evolution approach are in a good agreement with the LOD values calculated above (Figure 2). The abrupt decrease of average MRE increment can be clearly observed on the Figure 2B. Estimated LOD value is equal to 6 × 10^−4^ mol/L, which is close to the LOD_pu_ value.

### 4.2. Multisensor System for La Quantification in La/Y/Gd Mixtures

The experimental data (34 samples × 24 sensors) were not suitable for LOD interval assessment approach because of only two measurements performed for each sample, which is insufficient for reliable instrumental response variance calculation. Besides, the large number of sensors and the heteroscedasticity of their responses lead to major reduction of the sensor set for var(x) further calculation, which is undesirable. Nevertheless, LOD_pu_ value can be calculated from the PLS model built on the first three latent variables (Table 3).

In this case, the LOD value, estimated by the MRE evolution algorithm (3 × 10^−4^ mol/L), is lower than LOD_pu_, but the both values are still in a good agreement. 

### 4.3. Multisensor System for Determination of Urine Ionic Composition

**Figure 3:**
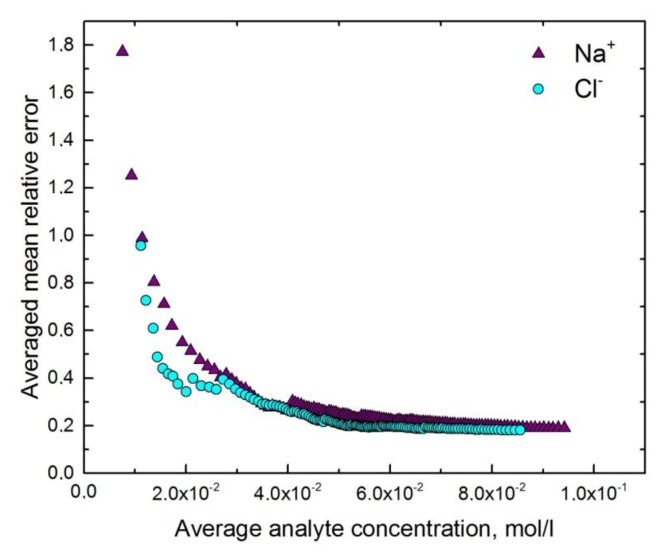
Performance visualization of the multisensor system, designed for determination of urine ionic composition. Violet triangles correspond to the PLS model built for Na^+^, blue circles—for Cl^−^.

As in the previous case, the variance of instrumental signal was difficult to calculate because of the large number both of the samples and the sensors (132 samples × 22 sensors). PLS models have been built for two chosen compounds (Na^+^, Cl^−^) on the three first LVs. The results of PLS model cross-validation and LOD_pu_ values are shown in the Table 3. Since Na^+^ and Cl^−^ concentrations are in the same range, it is possible to compare their estimated LOD values looking directly on the graph (Figure 3). The more abrupt is the transition from high MRE values to low ones, the better is the PLS model performance.

The estimated LOD value for Na^+^ and Cl^−^ is in the range of 3–4 × 10^−2^ mol/L (Table 4). It is half the calculated LOD_pu_ values, as well as in the previous case. It might be explained by moderate quality of the built model and, consequently, large variance of the residuals, which effects on the LOD_pu_ value.

## 5. Conclusions

The proposed methodology can be considered as a simple and intuitive approach to LOD estimation for multisensor systems. The better is a regression model quality and the more homoscedastic are the instrumental responses; the more satisfying is the agreement between the estimated LOD and the LOD calculated according to more theoretically strict approaches (for example, pseudo-univariate LOD). Nevertheless, in all cases mentioned in this work, the difference between the estimated and pseudo-univariate LOD values do not exceed 3 times. The MRE evolution approach does not require some additional measurements or calculations, but it is recommended to fulfill the following requirements:

- the PLS model (or any other regression model) has to be optimized;
- sample concentration values should uniformly cover the concentration range.

The proposed approach allows for comparison between different multisensor systems, applied for the same task, or for comparison of LODs for several analytes determined with the single multisensor system. The graphical representation of a multisensor system functioning in the whole range of the given concentration set might be useful as a visual illustration of its performance.

## Figures and Tables

**Table 1:** Characteristics of the multisensor systems under study.

Multisensor System	Composition of the Solution under Study	Analyte	Concentration Range, mmol/L	Number of Sensors
**1**	Ca/Mg + Na (background)	Ca^2+^	0.1–9.9	10
**2**	La/Y/Gd	La^3+^	0.01–1	24
**3**	Urine	Na^+^	3.8–255.5	22
		Cl^−^	11.1–22.3	

**Table 2:** Partial Least Squares (PLS) model characteristics and calculated limit of detection (LOD) values (mol/L) for the multisensor system #1. RMSECV: root-mean square error of cross-validation.

Analyte	PLS Model Characteristics			LOD Calculation, mol/L		
	Slope	R^2^	RMSECV	LOD_min_	LOD_max_	LOD_pu_
Ca^2+^	0.90	0.89	7.27 × 10^−4^	2.5 × 10^−4^	3.3 × 10^−4^	4.5 × 10^−4^

**Table 3:** PLS model characteristics and calculated LOD value (mol/L) for the La/Y/Gd multisensor system and the multisensor system designed for determination of urine ionic composition.

Object of Analysis	Analyte	PLS Model Characteristics			LOD Calculation
		Slope	R^2^	RMSECV	LOD_pu_, mol/L
**La/Y/Gd**	La^3+^	0.92	0.91	9.02 × 10^−5^	4.4 × 10^−4^
**Urine**	Na^+^	0.83	0.83	2.17 × 10^−2^	8.0 × 10^−2^
	Cl^−^	0.83	0.83	1.93 × 10^−2^	6.2 × 10^−2^

**Table 4:** Comparison of pseudounivariate and estimated by mean relative error evolution approach LOD values for all data sets.

Analyte	LOD_pu_, mol/L	Estimated LOD, mol/L
Ca^2+^	4.5 × 10^−4^	6 × 10^−4^
La^3+^	4.4 × 10^−4^	3 × 10^−4^
Na^+^	8.0 × 10^−2^	4 × 10^−2^
Cl^−^	6.2 × 10^−2^	3 × 10^−2^

## Supplementary Materials

The following are available online at https://www.mdpi.com/1424-8220/19/6/1359/s1, S1: LOD estimation function, S2: CaMgNa multisensor system.
