# Supplementary material for: A Simple Procedure to Assess Limit of Detection for Multisensor Systems
Source: Sensors (Basel). 2019 Mar 18;19(6):1359. doi: 10.3390/s19061359 (PMC6472210; doi:10.3390/s19061359)
Supplement: Supplementary file 1 [file sensors-19-01359-s001.zip › S1 LOD estimation function.pdf]

Supplementary material 1. MATLAB script for LOD estimation algorithm

```
function [lod_value] = lodestimation(data,k)

%This function allows to estimate a limit of detection value
%for a given regression model.
%
%Input arguments
%
%data - a matrix Nx2, where N is a number of samples, the first column
%contains measured concentration values (obtained by a reference method),
%the second one - concentrations, predicted by a regression model.
%NB!
%Verify if the both columns have the same length, the concentration
%values are in the same molar units (e.g., mol/l, cmol/l) and are sorted
%in ascending order!
%
%k - the accuracy criterion (defines the acceptable magnitude of
%mean relative error increment fluctuations around zero in %).
%Choose 1 for realistic LOD estimation, the higher is k, the more
%optimistic are the results.
%
%Output values
%
%lod_value - an estimated LOD value. Round to nearest integer is
%recommended.
%
%figure "System Functioning" is a visualisation of the system performance
%on different concentration ranges.
%
%figure "LOD estmation" is a graphical representation of LOD estimation.
%The horizontal line corresponds to the chosen k value, the vertical line
%corresponds to the LOD value.

ymeas = data(:,1);
ypred = data(:,2);
ymeas_l = length (ymeas);
mre = zeros(ymeas_l,1);

     for i = 1:ymeas_l
         mre(i,1) = abs((ymeas(i,1) - ypred(i,1))/ymeas(i,1));
     end

     averaged_mre = zeros(ymeas_l-1,1);
     averaged_conc = zeros(ymeas_l-1,1);

     for i = 1:ymeas_l-1

         averaged_mre (i,1) = abs(mean(mre(1:i+1,1)));
         averaged_conc (i,1) = abs(mean(ymeas(1:i+1,1)));

     end

     incr_mre = zeros(ymeas_l-2,1);

      for i = 1:ymeas_l-2
```

```
        incr_mre (i,1) = abs(averaged_mre(i+1,1) - averaged_mre(i,1))*100;
     end

    lod_crit = ones(ymeas_l-2,1)*k;

    lod_incr = find(round(incr_mre)>k, 1, 'last' );
     if isempty(lod_incr)

         error('LOD value is out of the given concentration range');

     else

         lod_value = averaged_conc(lod_incr+1,1);
         lod_line = ones(ymeas_l-2,1)*lod_value;

     end

   figure('Name','System Functioning');

plot(averaged_conc,averaged_mre,'LineStyle','none','Marker','o','MarkerEdgeColor
','black','MarkerFaceColor','r')
   title('System Functioning')
   xlabel('Averaged concentration, M')
   ylabel('Averaged MRE');
   figure('Name','LOD estimation');
   plot(averaged_conc(1:ymeas_l-
2,1),incr_mre,'LineStyle','none','Marker','o','MarkerEdgeColor','black','MarkerF
aceColor','r')
   title('LOD estimation')
   xlabel('Averaged concentration, M')
   ylabel('Averaged MRE increment,%')
   hold on
   plot(averaged_conc(1:ymeas_l-2,1),lod_crit,'-g')
   hold on
   plot(lod_line,incr_mre,'-g');
```
