# Supplementary material for: A Simple Procedure to Assess Limit of Detection for Multisensor Systems
Source: Sensors (Basel). 2019 Mar 18;19(6):1359. doi: 10.3390/s19061359 (PMC6472210; doi:10.3390/s19061359)
Supplement: Supplementary file 1 [file sensors-19-01359-s001.zip › S2 CaMgNa multisensor system.pdf]

Supplementary Material 2. Description of Ca/Mg/NA multisensor system

**Reagents**

High molecular weight poly(vinyl chloride) (PVC), 2-nitrophenyl octyl ether (NPOE), (bis-(2-ethylhexyl) sebacate (DOS), 2-fluorophenyl-2-nitrodiphenyl ether (FNDPE), tris-(2-ethylhexyl) phosphate (TOP), bis(2-ethylhexyl) phthalate (DOP), tetrakis-(trifluoromethyl)phenyl borate (KTTFMPB), Calcium ionophore**I**([(−)-(R,R)-N,N′-bis-[11-(ethoxycarbonyl)undecyl]-N,N′-4,5-tetramethyl-3,6-dioxaoctanediamide]) and Magnesium ionophore **I** (N,N′-Diheptyl-N,N′-dimethyl-1,4-butanediamide), were purchased from Fluka (Switzerland). Chlorinated cobalt dicarbollide (CCD) was obtained from Katchem (Czech Republic) as a cesium salt and then converted to the acidic ($H^+$) form prior to the membrane preparation.

NaCl, $CaCl_2$, $MgCl_2$ metal chloride salts were purchased from Vekton (Russia, analytical grade). Tetrahydrofurane (THF) purchased from Merck (Germany) was freshly distilled before use.

**Membrane preparation**

The ionophore-free polymeric membranes consisted of 33 wt% PVC, 66 % plasticizer and 1 % of a lipophilic additive. All ten compositions studied in this paper are described in details in the Table 1. To prepare the membrane, the components (300 mg in total) were dissolved in 3 mL of THF, poured into a Teflon cylindrical vessel and left to dry for 48 h. Then membranes of 7 mm in diameter (three of each composition) were cut from the resulting film and glued to a PVC tube body.

**Potentiometric measurements**

The sensor set comprised 30 cross-sensitive cationic sensors (10 types, 3 pieces of each kind).

Electrochemical measurements were carried out in the following galvanic cell:

Cu | Ag ⁞ AgCl, $KCl_{sat}$ |sample solution | membrane | NaCl 0.01 M |AgCl ⁞ Ag | Cu

The EMF measurements were performed against the standard reference Ag/AgCl (ZIP, Belorussia) electrode at room temperature (20 $^oC$) using a multi-channel digital mV-meter with high input impedance (Sensor Systems, Russia). The potential values were recorded with the 0.1 mV precision for 3 minutes in 10 sec intervals. The results were presented as an average of the last 3 EMF values during these 3 minutes.

The glass pH electrode (ZIP, Belorussia) was used for pH control.

Activity coefficients were calculated according to Debye–Hükkel theory.

Calibration of the sensors was performed in chloride salts $Ca^{2+}$- and $Mg^{2+}$ solutions in the concentration range between $10^{-7}$ and $10^{-2}$ M. The sensor sensitivity was calculated as a slope of the electrode response (mV per decade) for the metal ion concentration range between $10^{-4}$ and $10^{-2}$ M. All calculated slopes were averaged over the three replica sensors of each composition and multiple replicated measurements.

To assess the correlation between the array response and the Ca2+ and Mg2+ concentration, the calibration was performed for the sample set containing 40 binary Ca-Mg mixtures (Set 1).

Table 1. Composition of the membranes

| Name | Plasticizer | Lipophilic salt |
|---|---|---|
| 1 | 2-nitrophenyl octyl ether (NPOE) | Tetrakis(trifluoromethyl)phenyl borate (KTTFMPB) |
| 2 | 2-nitrophenyl octyl ether (NPOE) | chlorinated cobalt dicarbollide (CCD) |
| 3 | di(2-ethylhexyl) sebacate (DOS) | Tetrakis(trifluoromethyl)phenyl borate (KTTFMPB) |
| 4 | di(2-ethylhexyl) sebacate (DOS) | chlorinated cobalt dicarbollide (CCD) |
| 5 | 2-fluorophenyl-2-nitrodiphenyl ether (FNDPE) | Tetrakis(trifluoromethyl)phenyl borate (KTTFMPB) |
| 6 | 2-fluorophenyl-2-nitrodiphenyl ether (FNDPE) | chlorinated cobalt dicarbollide (CCD) |
| 7 | tris-(2-ethylhexyl) phosphate (TEHP) | Tetrakis(trifluoromethyl)phenyl borate (KTTFMPB) |
| 8 | tris-(2-ethylhexyl) phosphate (TEHP) | chlorinated cobalt dicarbollide (CCD) |
| 9 | bis(2-ethylhexyl) phthalate (DOP) | Tetrakis(trifluoromethyl)phenyl borate (KTTFMPB) |
| 10 | bis(2-ethylhexyl) phthalate (DOP) | chlorinated cobalt dicarbollide (CCD) |
